# Seroprevalence of Rift Valley fever virus in livestock during inter-epidemic period in Egypt, 2014/15

**DOI:** 10.1186/s12917-017-0993-8

**Published:** 2017-04-05

**Authors:** Claudia Mroz, Mayada Gwida, Maged El-Ashker, Mohamed El-Diasty, Mohamed El-Beskawy, Ute Ziegler, Martin Eiden, Martin H. Groschup

**Affiliations:** 1 Institute of Novel and Emerging Infectious Diseases, Friedrich-Loeffler-Institut, Südufer 10, 17493 Greifswald - Isle of Riems, Germany; 2 Department of Hygiene and Zoonoses, Faculty of Veterinary Medicine, Mansoura University, Mansoura, 35516 Egypt; 3 Department of Internal Medicine and Infectious Diseases, Faculty of Veterinary Medicine, Mansoura University, Mansoura, 35516 Egypt; 4 Animal Health Research Institute-Mansoura Provincial Laboratory, Mansoura, Egypt; 5 Faculty of Veterinary Medicine, Mansoura University, Mansoura, Egypt

**Keywords:** Rift Valley fever virus, Livestock, Inter-epidemic period, Surveillance, Egypt

## Abstract

**Background:**

Rift Valley fever virus (RVFV) caused several outbreaks throughout the African continent and the Arabian Peninsula posing significant threat to human and animal health. In Egypt the first and most important Rift Valley fever epidemic occurred during 1977/78 with a multitude of infected humans and huge economic losses in livestock. After this major outbreak, RVF epidemics re-occurred in irregular intervals between 1993 and 2003. Seroprevalence of anti-RVFV antibodies in livestock during inter-epidemic periods can be used for supporting the evaluation of the present risk exposure for animal and public health. A serosurvey was conducted during 2014/2015 in non-vaccinated livestock including camels, sheep, goats and buffalos in different areas of the Nile River Delta as well as the furthermost southeast of Egypt to investigate the presence of anti-RVFV antibodies for further evaluating of the risk exposure for animal and human health. All animals integrated in this study were born after the last Egyptian RVF epidemic in 2003 and sampled buffalos and small ruminants were not imported from other endemic countries.

**Results:**

A total of 873 serum samples from apparently healthy animals from different host species (camels: *n* = 221; sheep: *n* = 438; goats: *n* = 26; buffalo: *n* = 188) were tested serologically using RVFV competition ELISA, virus neutralization test and/or an indirect immunofluorescence assay, depending on available serum volume. Sera were assessed positive when virus neutralization test alone or least two assays produced consistent positive results. The overall seroprevalence was 2.29% (95%CI: 1.51–3.07) ranging from 0% in goats, 0.46% in sheep (95%CI: 0.41–0.5), and 3.17% in camels (95%CI: 0.86–5.48) up to 5.85% in buffalos (95%CI: 2.75–8.95).

**Conclusion:**

Our findings assume currently low level of circulating virus in the investigated areas and suggest minor indication for a new RVF epidemic. Further the results may indicate that during long inter-epidemic periods, maintenance of the virus occur in vectors and also most probably in buffaloes within cryptic cycle where sporadic, small and local epidemics may occur. Therefore, comprehensive and well-designed surveillance activities are urgently needed to detect first evidence for transition from endemic to epidemic cycle.

**Electronic supplementary material:**

The online version of this article (doi:10.1186/s12917-017-0993-8) contains supplementary material, which is available to authorized users.

## Background

Rift Valley fever is a mosquito-borne zoonotic disease in ruminants, camels and humans caused by Rift Valley fever virus (RVFV), a Phlebovirus within the family *Bunyaviridae* [1, 2]. The viral disease was identified for the first time in 1930 in Kenya and is characterized by high fever and abortion in livestock and high neonatal mortality mainly in sheep [3–6]. Infected humans show a mild febrile illness, however in 1–2% of cases the patients develop severe complications such as ocular disease, hemorrhagic fever syndrome or encephalitis [7]. Typically the general case fatality is low (1–3%). But patients with hemorrhagic fever syndrome show fatality rates up to 50% [8].

It has been reported that more than 30 mosquito species from 6 genera can transmit the virus to susceptible hosts [7]. Bites of infected mosquitos play the most important role for ruminant infection [7, 9]. The direct contact with infectious materials when handling with sick or dead infected animals, abortion material or other fresh tissues represents the main transmission route in humans. Due to climatic changes and high level livestock trade, the virus is widespread in Africa and spread also in 2000 to Saudi Arabia and Yemen [5, 9, 10]. Climatic and environmental conditions like heavy rainfalls with increasing mosquito population redound consistently to new RVF outbreaks. Severe outbreaks occurred for instance in Mauritania and in South Africa in 2010, in Kenya, Tanzania and Somalia in 2007 as well as in Sudan in 2008 and 2010 [11–14].

The RVFV was introduced to Egypt in 1977 and caused an extensive epidemic with thousands of infected humans, more than 600 deaths and high economic losses in livestock affecting five governorates in the Nile Delta (Sharqia, Aswan, Qalyubia, Giza and Assiut [5, 15–18]. Up to now, it has been considered the major outbreak for Egypt and one of the largest epidemics in the RVF history of Africa. After a long inter-epidemic period, the RVF re-occurred in the Nile Delta of Egypt in 1993 in Aswan and Damietta governorates [19–21]. Further outbreaks recurred in 1994 (Beheira and Kafr el Sheikh governorates) as well as in 1997 (Assuit and Aswan governorates) and most recently in 2003 (Kafr el Sheikh governorate) [19, 21–23]. The sources of the diverse outbreaks are broadly discussed but the maintenance of the virus during inter-epidemic periods is still poorly understood [21, 24]. It has been reported that the presence of unvaccinated susceptible livestock in combination with favorable conditions for mosquito breeding and spread are facilitating conditions for the persistence of the RVFV in Egypt [21]. Detection of RVFV specific antibodies in non-immunized animals a long time after the last RVF epidemic indicates endemic maintenance of the virus in inter-epidemic periods and seroconversion often occurs without any clinical signs in the livestock population [25, 26].

Evidence of circulating virus in the current inter-epidemic phase has been found by Ramadan [27] in 2009 who proves the presence of anti-RVFV-antibodies in Dakahlia governorate in different livestock species (sheep = 20%, goats = 17%, cattle = 5% and buffalos = 11% respectively). An additional survey from Marawan [28] in 2012 shows related prevalence rates in non-immunized sheep, goats, camels, cattle and buffalos (17, 7, 0, 19 and 10%, respectively) in four governorates in the Nile delta of Egypt (Qalyubia, Dakahlia, Sharkia, Kafr El Sheikh). A compilation of outbreak sites and sites of previous seroepidemiological studies in egypt are indictaed in Additional file 1.

Seroepidemiological studies could merely give a brief insight into the infection status for a short period in which the study was carried out. Therefore, the need for continuous inspections of the antibody prevalence in susceptible species is highly recommended in endemic areas. This paper presents an overview about the antibody presence in non-vaccinated susceptible hosts including sheep, goats and buffalos from Dakahlia governorate, a vulnerable part for new RVF epidemic in Egypt. Furthermore camel sera from an abattoir near Cairo and from southeast of Egypt, near the border to Sudan, were investigated. Aim of this study was to determine the current status of anti-RVFV antibodies in non-immunized hosts for further evaluating the exposure risk for animal and human health.

## Methods

### Animal population, study areas and sample collection

**Fig. 1:**
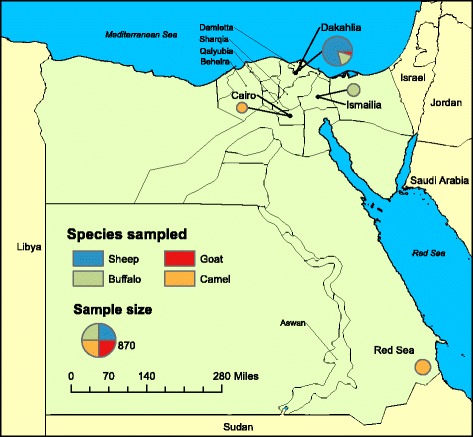
Region of samples collection. Samples from small ruminants and buffalos from small holders were collected in Dakahlia governorate. Further buffalo samples, derived from farm animals were collected from Ismailia governorate. Camel derived sera were provided from an abattoir near Cairo and from Rea Sea governorate. Previous Egyptian RVFV outbreaks occurred in indicated governorates (Sharqia, Aswan, Qalyubia, Damietta and Beheira)

The present seroepidemiological study included a total of 873 non-immunized, apparently healthy animals including small ruminants, buffalos and camels, which were sampled during 2014 to 2015 in different areas of Egypt. Due to the high susceptibility of sheep and their suitability for the use as sentinel animals [9, 29–31], 438 sheep samples from 8 different small holding herds were collected during 2015 (Table 1). All animals were settled in Dakahlia governorate, a central part of the Nile River Delta, in open yard holdings, with movement restrictions in the vicinity (Fig. 1). The number of animals in the flocks varied from 20 to 87 animals (mean (M) = 54.75; standard deviation (SD) =23.24). Additionally sheep herd 1 and 2 included a total of 26 goats (17 in herd 1 and 9 in herd 2). Small holders were characterized by keeping less than 50 animals, often in family farming with poor resources, no health management and prevalently with more than one species.

**Table 1:** Samples ordered by species, holding system, and region

Species	Holding	Herd number	Age	Region	Number of samples	Date of sample collection
Sheep	Open yard (small holder)	1	2–5	Dakahlia Governorate (Aga district)	87	April 2015
Goat			2–3		17	
Sheep	Open yard (small holder)	2	2–10	Dakahlia Governorate (Aga district)	82	May 2015
Goat					9	
Sheep	Open yard (small holder)	3	2–5	Dakahlia Governorate (Belkas district)	50	May 2015
Sheep	Open yard (small holder)	4	1–4	Dakahlia Governorate	78	June 2015
Sheep	Open yard (small holder)	5	1–5	Dakahlia Governorate	40	June 2015
Sheep	Open yard (small holder)	6	2–4	Dakahlia Governorate	49	June 2015
Sheep	Open yard (small holder)	7	3–7	Dakahlia Governorate	32	June 2015
Sheep	Open yard (small holder)	8	2–4	Dakahlia Governorate	20	February 2015
Buffalo	Small holder	1	3–7	Dakahlia Governorate (Belkas district)	31	2014
Buffalo	Small holder	2	3–7	Dakahlia Governorate	9	2014
Buffalo	Small holder	3	3–7	Dakahlia Governorate	21	2014
Buffalo	Small holder	2	3–5	Dakahlia Governorate (Belkas district)	27	2015
Buffalo	Farm	1	4–6	Ismailia Governorate	100	2015
Camel	Abattoir (El Basatine)		5–7	Cairo	71	2014
Camel	Imported from Sudan		2–7	Red Sea Governorate (Halayb and Shalatein)	150	2015

Further ruminant samples were collected from 188 Asian water buffalos (Table 1, Fig. 1). 88 serum samples derived from four small holdings located in Dakahlia governorate (61 samples collected in 2014 and 27 samples in 2015). Small holder flocks included 9 to 31 buffalos (M = 22; SD = 8.3). Additional 100 samples were collected from buffalos from a farm in Ismailia governorate, situated in the south eastern of the Nile Delta at the Suez Canal. In general farms are characterized by herd sizes over 50 animals and a good health management and restricted movements of the animals (only to local markets in the country). In this study the sampled farm kept 1200 animals. All buffalos and small ruminants included in this study were born after the last Egyptian RVF epidemic in 2003 and were not imported from other endemic countries.

To investigate the role of camels in Egypt additional samples were collected from this species. Most of the Egyptian camels were imported from Sudan and were kept in quarantine near the border of Sudan in Red Sea governorate after importation (Fig. 1). We investigated 150 of those camel derived sera and additional 71 camel sera taken at an abattoir near Cairo (Table 1).

All blood samples were randomly collected from apparently healthy animals and an informed consent for RVFV investigation was given by the owners. All procedures were performed in accordance with the principles and specific guidelines presented in the Guidelines for the Care and Use of Agricultural Animals in Research and Teaching, 3^rd^ ed. [32].

Animal specimens were collected under the direction of the Mansoura University, Mansoura, Egypt, within the framework of the project ‘Brucellosis, Q-fever and viral hemorrhagic fever infections in Egypt’. The collected blood samples were kept overnight at room temperature to allow blood clotting. On the next day, clear sera were collected and stored at −20 °C until shipping. In Germany, the sera were subjected to Gamma radiation (Synergy Health, Radeberg) and were irradiated for 24 h by 30 k gray. This treatment was carried out to ensure that the examinations can be done under BSL 2 conditions. After radiation, the sera were stored at −20 °C until serological examinations.

### Serological testing’s

#### Enzyme linked immunosorbent assay (ELISA)

Each sample was first analyzed with the commercial ID Screen® RVFV competition multispecies ELISA (ID VET, Montpellier, France) according to the manufacturers’ instructions [30]. The cELISA is based on recombinant nucleoprotein and detects anti-RVFV antibodies (both IgM and IgG).

#### Virus neutralization test (VNT)

Investigation of neutralizing antibodies of the samples was carried out by VNT, the gold standard for serological RVF analysis. VNT was performed using the RVFV-MP12 vaccine strain according to the OIE terrestrial manual, 2015 [33]. Briefly, each serum samples were run in duplicate and titrated in three steps (1:5, 1:10, 1:20). The diluted virus (100 TCID_50_ per well) was added to 1:10 and 1:20 serum dilution, 1:5 served as serum control. After 30 min incubation at 37 °C and 5% CO_2_ 3×10^5^ Vero 76 cells (Collection of Cell Lines in Veterinary Medicine, Friedrich-Loeffler-Institut, Germany) per ml were added to each well. Positive and negative control sera as well as cell and virus controls were included in each test cycle. After six days at 37 °C and 5% CO_2_ cytopathic effects were revealed and the plates were fixed with formalin, colored with crystal violet and the VNT titer was calculated. According to the Behrens-Kaerber method the neutralizing antibody titer of the samples was defined as the 50% neutralization dose (ND_50_). Serum samples with ND_50_ values above 10 were determined as positive; samples with lower titer than 10 were determined as negative. While the endpoint titer was not in focus of interest all sera with a higher ND_50_ value than 30 were not further diluted and were summarized as ≥30.

#### Indirect Immunofluorescence assay (IIFA)

Additional confirmation of positive and inconclusive results in previous tests was performed with an in-house immunofluorescence assay (IIFA) according to a previously published protocol [11]. In short diluted serum samples were added to commercial RVFV immunofluorescence slides from Euroimmun (Lübeck, Germany). After 30 min reaction time, the slides were washed and the conjugate was added. A donkey anti-sheep IgG Cy3 (Indocarbocyanine)-labelled antibody (Dianova, Hamburg, Germany) was used for small ruminants and a goat anti-bovine IgG Cy3-labelled antibody (Dianova) for buffalo samples, respectively. A polyclonal rabbit-anti-camel antiserum (Bethyl laboratories, Montgomery, TX, USA) was used as a second antibody for camel samples followed by a Cy3-conjugated goat anti-rabbit antibody (Dianova) according to the method described by Jäckel [11]. The slides were evaluated with a fluorescence microscope (Nikon, Tokyo, Japan).

#### IgM ELISA

To detect recent infection samples showing positive cELISA results (positive to IgG or IgM) in combination with negative IIFA results (detects only IgG) were further tested with the ID Screen® Rift Valley Fever IgM capture ELISA (ID VET, Montpellier, France) according to the manufacturer’s instructions.

### Interpretation of the results

Sera were classified as ‘positive’ when VNT alone or at least two assays produced consistent positive results. In a limited number of cases, due to low sample volume, application of combined analysis was not possible. Referred to the required serum volume for performing the different test systems, such sera were first tested by IIFA and then, if possible, confirmed by VNT and/or cELISA. If only one test could be carried out, the result of this test was used for classification. Combined test performance with result conclusion was considered to increase the total test accuracy. Positive results indicated seroconversion of the animals after contact to the antigen, independent of the point of exposure.

The true prevalence of each species/herds was calculated using the calculation tool of ‘Working in Epidemiology’ from the University of Zaragoza [20] (http://www.winepi.net/uk/index.htm).

## Results

Antibodies against Rift Valley fever virus were detected with three different methods, an approach, which enabled the identification of antibodies against the nucleoprotein in case of competition ELISA, neutralizing antibodies (frequently against the glycoprotein Gn) in case of the VNT and of IIFA reactive antibodies.

Only two out of 438 sheep samples were tested positive displaying a seroprevalence of 0.46% (95% confidence interval [95%CI:] 0.414–0.5). In addition no seropositive goat was detected. Results from individual tests as well as the data interpretation are shown in Table 2. A detailed analysis of positive samples is shown in Table 3.

**Table 2:** Serological analysis of Egyptian serum samples from small ruminants, buffalos and camels with cELISA (ID Vet Screen Rift Valley Fever competition ELISA (multispecies), indirect immunofluorescence assay (IIFA) and virus neutralization test (VNT) (A) Results grouped into species. Result conclusion (B). Detailed prevalence of herds is shown in part (C)

(A)											
	ID screen® multispecies competition ELISA				Virus neutralization test				Immunofluorescence		
Species	Tested	Positive	Doubtful	Negative	Tested	Positive	Negative	Tested	Positive	Doubtful	Negative
Sheep	417	3	1	413	381	0	381	88	3	3	82
Goat	26	0	0	26	26	0	26	n.t.	n.t.	n.t.	n.t.
Buffalo (small holder)	78	8	0	70	56	2	54	32	8	0	24
Buffalo (farms)	95	1	0	94	88	0	88	16	0	0	16
Camels abattoir	1	1	0	0	59	6	53	71	2	0	69
Camels imported from Sudan	130	0	0	130	121	0	121	20	0	0	20
Total:	747	13	1	733	731	8	723	227	13	3	211
(B)											
Result conclusion	animals in the herds	tested	positive	inconclusive	negative	prevalence (%)	95% CI				
Sheep	440	438	2	1	435	0.46	0.41–0.5				
Goat	17	26	0	0	26	0.00	0.00–0.00				
Buffalo (small holder)	91	88	11	0	77	12.5	11.25–13.75				
Buffalo (farm)	1200	100	0	0	100	0.00	0–2.83				
Came l (abattoir)		71	7	0	64	9.86	2.92–16.79				
Imported camel		150	0	0	150	0.00	0.00–0.00				
(C)											
Results in individual herds	animals in the herds	tested	positive	prevalence (%)	95% C I						
sheep herd 4	78	78	1	1.28	1.28–1.28						
sheep herd 5	42	40	1	2.5	1.44–3.56						
buffalo small holder 1	33	31	6	19.35	15.93–22.78						
buffalo small holder 2	9	9	2	22.22	22.22–22.22						
buffalo small holder 3	22	21	3	14.29	11.09–17.48

**Table 3:** Individual positive results of Egyptian serum samples from small ruminants, buffalos and camels with cELISA (ID Vet Screen Rift Valley Fever competition ELISA (multispecies), indirect immunofluorescence assay (IIFA) and virus neutralization test (VNT)

Sample number			Herd	Age (years)	ID vet cELISA S/N%	IIFA	VNT ND50 value*	*Result* *conclusion*
Sheep								
EG	12	15/OV	1	2–7	*32,82*	negative	negative	negative
EG	247	15/OV	4	1–4	*17,79*	++	negative	*positive*
EG	256	15/OV	4	1–4	*16,24*	negative	negative	negative
EG	282	15/OV	4	1–4	49,48	negative	negative	negative
EG	299	15/OV	5	1–5	*not tested*	+/−	negative	negative
EG	304	15/OV	5	1–5	*not tested*	+/−	negative	negative
EG	321	15/OV	5	1–5	*not tested*	+/−	*not tested*	*inconclusive*
EG	328	15/OV	5	1–5	*not tested*	+	*not tested*	*positive*
EG	394	15/OV	7	1–5	*not tested*	++	negative	*inconclusive*
Buffalo - small holder								
EG	4	14/BF	1	3–7	88,81	negative	*10*	*positive*
EG	9	14/BF	1	3–7	*4,90*	not tested	*not tested*	*positive*
EG	14	14/BF	1	3–7	66,06	negative	*20*	*positive*
EG	15	14/BF	1	3–7	*4,03*	++	*not tested*	*positive*
EG	20	14/BF	1	3–7	*3,88*	++(+)	*not tested*	*positive*
EG	23	14/BF	1	3–7	*3,65*	++	*not tested*	*positive*
EG	34	14/BF	2	3–7	*4,01*	++(+)	*not tested*	*positive*
EG	38	14/BF	2	3–7	*18,46*	++(+)	*not tested*	*positive*
EG	41	14/BF	3	3–7	*37,71*	+	*not tested*	*positive*
EG	42	14/BF	3	3–7	*10,88*	++	*not tested*	*positive*
EG	53	14/BF	4	3–7	*not tested*	(+)	*not tested*	*positive*
Buffalo - farms								
EG	59	15/BF	1	4–6	*8,80*	negative	negative	Negative
Camel - abattoir								
EG	3	14/CM	abattoir	5–7	*not tested*	negative	*10*	*positive*
EG	4	14/CM	abattoir	5–7	*not tested*	negative	*>30*	*positive*
EG	18	14/CM	abattoir	5–7	*not tested*	negative	*>30*	*positive*
EG	32	14/CM	abattoir	5–7	*5,76*	++	*>30*	*positive*
EG	46	14/CM	abattoir	5–7	*not tested*	negative	*>30*	*positive*
EG	58	14/CM	abattoir	5–7	*not tested*	++	*not tested*	*positive*
EG	67	14/CM	abattoir	5–7	*not tested*	negative	*>30*	*positive*

cELISA results with a percentage of inhibition lower than 40 were defined as positive, between 40 and 50% as inconclusive and results higher than a percentage of inhibition of 50 were defined as negative. IIFA results were defined from low (+) to strong (+++) straining, or as inconclusive (+/−). ND_50_ values lower than 10 were defined as negative when performing VNT. Titers higher than ND_50_ values of 20 indicate a strong immune response

Positive and inconclusive findings and results are indiciated in italics

Four sera were subjected to IgM capture ELISA to detect recent infections. All sera gave negative results in this test.

Buffalo derived sera gained from four small holders in Dakahlia governorate revealed that 11 out of 88 sera were determined positive corresponding to a seroprevalence of 12.5% (95% CI: 11.25–13.75) (Tables 2 and 3). Individual small holding herds showed prevalence of 19.35 (95%CI: 15.93–22.78), 22.22% (95%CI: 22.22–22.22), 14.29% (95%CI: 11.09–17.48) and 0%, respectively. In contrast, no positive results were observed in 100 buffalo sera from the farm. The overall seropositivity for 188 buffalo samples was therefore 5.85% (95% CI: 2.75–8.95).

Finally also camel sera were subjected to serological analysis. Seven out of 71 sera of animals from the slaughterhouse showed positive results (five of them displayed ND_50_ values of 30 or higher [Table 3]); however none of the samples from imported camels were determined positive. The corresponding prevalence for camels from abattoir was 9.86% (95% CI: 2.92–16.79) and 3.17% (95% CI: 0.86–5.48) comprised the total camel number. The total seroprevalence of all investigated animals (*n* = 873) was 2.29% (95% CI: 1.30–3.28).

## Discussion

The seroepidemiological study presented here provided insights into the current RVF antibody status in non-immunized livestock and enabled a preliminary evaluation of the exposure risk to the virus in susceptible host species.

Sheep are the most susceptible species to RVFV infections [9, 30] and often used as sentinel animals in endemic areas [29, 31]. Therefore the investigation of sheep samples in the Nile Delta, a vulnerable part of Egypt for new RVF epidemics was in the focus of this study. The presence of antibodies against RVFV in sheep samples was very low (0.46%). Studies conducted 7 years after the large epidemic/epizootic in 1977 encompassing 1714 sheep showed a prevalence of 1.2% in the governorate of Dakahlia [34]. These results are in line with the finding obtained in this study i.e. 12 years after the last RVF outbreak in Egypt which occurred in 2003.

Unlike to the results stated here, previous reports conducted in the present inter-epidemic period document a relatively higher seropositivity in small ruminants located in the Nile Delta of Egypt. Ramadan, [27] found in 2009 a prevalence of 19.9% (*n* = 183 sheep samples from Dakahlia governorate) and also the results of Marawan, [28] in year 2012 indicated a considerably higher positivity of 12.3% (*n* = 70 sheep samples from the same governorate). Further studies in additional Nile Delta governorates showed likewise higher prevalence levels (a total of 17.6% in Qalyubia, Sharkia and Kafr el Sheikh in 2012; 21.4% in El Monofia, Beheira and Kafr el Sheikh in 2009/2010 [28, 35]). Deviating prevalence levels in small ruminants examined in 2009 and 2012 as compared to our data for sheep in 2015 indicate that the more recent virus circulation was negligible. Nevertheless, as a) none of these animals was imported and b) animals were transported only locally, a RVFV circulation can be assumed, especially also since all animals were born after the last reported RVFV outbreak.

The significant role of camels in transmission and spread of RVFV was already demonstrated during the Egyptian RVF outbreak in 1997. Due to often rare clinical manifestations in camels and the assumption that camels are less susceptible, camels, imported from other endemic countries in particular can carry the virus [7, 36]. Egypt imports camels for human consumption from endemic countries like Sudan or the Horn of Africa [21]. None of the imported camels included in the here presented study showed a positive result, whereas the collected specimens from abattoir-camels gave a prevalence of 9.86%. Elevated neutralization titers in these camels might suggest an antigen contact quite recently. Unfortunately there was no further information about the history of the origin of the abattoir-camels. Therefore it could not be ruled out, that those animals were previously imported. Previous studies in Egyptian camels during the present inter-epidemic period are conducted in 2009/2010 with 10 camel sera from an abattoir [35] and in 2012 encompassing 100 camels from Qalyubia governorate [28]. Likewise no further information about the origin of the animals is given in these reports. Both studies detected no antibodies in Egyptian camels. Only 10% of imported animals are tested against RVFV antibodies directly after the import as a routine disease control. Positive results in camels in this study were supposed to be imported positive animals from other endemic countries. The risk to import viremic animals should not be neglected; hence continuing investigations on the role of camels should be in focus of further investigations.

Buffalos presumably play a role as amplifying hosts during inter-epidemic periods. Antibodies against RVFV were detected in several studies in different African countries during inter-epidemic times (e.g. prevalence rates of 21, 15.6 and 12.5% in South Africa, Kenya and Botswana respectively [37–39]).

The analyzed buffalo sera in this study showed also a relatively slight prevalence of 5.85%. This finding corresponds to results obtained from Horton in 2009 [35], which included 153 buffaloes from the Muneeb abattoir in central Egypt, who reported a prevalence of 3% and also to the results from Marawan, 2012, [28] who obtained a prevalence of 9.8% in 102 buffalo sera. Interestingly, there was a striking difference of the prevalence levels for different holding systems: it ranged from 0% in buffalo on the farm compared to animals owned by small holders (12.5%). Individual small buffalo holdings showed seroprevalences of up to 22%, which might be due to a lower health status of the animals in small holding flocks. Additionally, farmed buffalos were located in Ismailia governorate. Ismailia is located marginal to the east lower Nile Delta, which could be a reason for lower presence of transmission of the virus to naïve animals.

Main vectors for the RVFV transmission in Egypt are mosquitos of genus Culex [40]. In 2009 Ramadan [27] collected these mosquitos from Dakahlia governorate and found 11.1% positive encompassing 806 mosquitos in 13 pools which indicates virus maintenance in the country. Due to more elaborate health management measures in larger farms, which might include also vector control programs, natural RVFV infections by Culex mosquitos in these farms are less likely as compared to small holder husbandry. We found a prevalence of 18% in buffalos sampled in 2014 (*n* = 61), whereas the small holder buffalos from 2015 (*n* = 27) showed only negative results. It would be of interest, to study in more detail, to which extent differences in the holding systems can influence the general infection risk for the animals. According to the farmer’s documentation the buffalos were not imported from other endemic countries. Moreover buffalos were restricted in their movements and were only transported to local markets. Therefore also the data obtained for buffalos (similar to those found in small ruminants) suggests an active virus transmission in Dakahlia governorate during the present inter-epidemic period in Egypt.

Altogether, a comprehensive and well-designed surveillance program allows the early detection of first indications for the transition from endemic to epidemic cycle. Such a surveillance program should include investigations on the serological status of all susceptible animals in areas at risk, the monitoring of vectors and the intensification of import controls for animals coming from endemic countries.

## Conclusions

The examination of 873 sera collected from sheep, goats, camels and buffalos gave an insight into the anti-RVFV antibody situation in Egypt during 2014/2015. All animals included in this study were born after the last Egyptian RVF epidemic in 2003 and, as to farmer’s records, buffalos and sheep were not imported from other endemic countries. Therefore the antibody prevalence observed in buffalos and sheep were results of cryptic virus transmissions during the present inter-epidemic period. Based on a general low prevalence in all investigated animal species, a currently low level of circulating virus in the investigated areas can be assumed. Due to the general lack of detailed data about the role of camels for cryptic virus transmissions in Egypt further investigations are needed.

Assuming the high susceptibility of small ruminants to RVF, our data indicate, that small ruminants in Egypt are not the main source of inter-epidemic virus circulation, which means infections of alternative animal hosts that are less receptive to clinical manifestations.

## Additional file

Additional file 1: Table S1. Comparison of outbreak sites and sites of previous seroepidemiological studies in Egypt. (DOC 18.7 kb)
