# Supplementary material for: Seroprevalence of Rift Valley fever virus in livestock during inter-epidemic period in Egypt, 2014/15
Source: BMC Vet Res. 2017 Apr 5;13:87. doi: 10.1186/s12917-017-0993-8 (PMC5382484; doi:10.1186/s12917-017-0993-8)
Supplement: Additional file 1: Table S1. — Comparison of outbreak sites and sites of previous seroepidemiological studies in Egypt. (DOC 18.7 kb) [file 12917_2017_993_MOESM1_ESM.doc]

**Additional file 1: Table S1**: Comparison of outbreak sites and sites of previous seroepidemiological studies.
